# 

**Published:** 1877-02

**Authors:** 


CONTENTS.

| | Page | | Page |
|---|---|---|---|
| Disease and Benevolence | 33 | Equanimity of Mind | 47 |
| Book Notices | 34 | A Sick Mind | 48 |
| Insanity | 35 | Keep Your Mouth Shut | 50 |
| Civilization and Health | 38 | Clerical Exposures | 51 |
| Consumption Cure | 43 | Out-Door Safety | 52 |
| Practical Knowledge | 44 | Blacking Boots | 53 |
| Why? | 46 | Heights | 53 |

